# Circulating microRNA-based screening tool for breast cancer

**DOI:** 10.18632/oncotarget.6786

**Published:** 2015-12-29

**Authors:** Pierre Frères, Stéphane Wenric, Meriem Boukerroucha, Corinne Fasquelle, Jérôme Thiry, Nicolas Bovy, Ingrid Struman, Pierre Geurts, Joëlle Collignon, Hélène Schroeder, Frédéric Kridelka, Eric Lifrange, Véronique Jossa, Vincent Bours, Claire Josse, Guy Jerusalem

**Affiliations:** ^1^ University Hospital (CHU), Department of Medical Oncology, Liège, Belgium; ^2^ University of Liège, GIGA-Research, Laboratory of Human Genetics, Liège, Belgium; ^3^ University of Liège, GIGA-Research, Laboratory of Molecular Angiogenesis, Liège, Belgium; ^4^ University of Liège, GIGA-Research, Department of EE and CS, Liège, Belgium; ^5^ University Hospital (CHU), Department of Gynecology, Liège, Belgium; ^6^ University Hospital (CHU), Department of Senology, Liège, Belgium; ^7^ Clinique Saint-Vincent (CHC), Department of Pathology, Liège, Belgium

**Keywords:** breast cancer, circulating microRNAs, biomarkers, minimally invasive screening

## Abstract

Circulating microRNAs (miRNAs) are increasingly recognized as powerful biomarkers in several pathologies, including breast cancer. Here, their plasmatic levels were measured to be used as an alternative screening procedure to mammography for breast cancer diagnosis.

A plasma miRNA profile was determined by RT-qPCR in a cohort of 378 women. A diagnostic model was designed based on the expression of 8 miRNAs measured first in a profiling cohort composed of 41 primary breast cancers and 45 controls, and further validated in diverse cohorts composed of 108 primary breast cancers, 88 controls, 35 breast cancers in remission, 31 metastatic breast cancers and 30 gynecologic tumors.

A receiver operating characteristic curve derived from the 8-miRNA random forest based diagnostic tool exhibited an area under the curve of 0.81. The accuracy of the diagnostic tool remained unchanged considering age and tumor stage. The miRNA signature correctly identified patients with metastatic breast cancer. The use of the classification model on cohorts of patients with breast cancers in remission and with gynecologic cancers yielded prediction distributions similar to that of the control group.

Using a multivariate supervised learning method and a set of 8 circulating miRNAs, we designed an accurate, minimally invasive screening tool for breast cancer.

## INTRODUCTION

Breast cancer is the most frequently diagnosed cancer in females worldwide; its rate in Western countries has increased since the 1990s [1]. During the same period, mortality from breast cancer has decreased due to early detection and improved treatments [2].

Currently, mammographic screening, followed by invasive core needle biopsies in cases of suspected malignancy, allows early breast cancer diagnosis. Mammographic screening is an accessible but unpleasant and inaccurate test; in 1000 screened women, 15 of these women are estimated to have a biopsy because of a suspicious abnormality, and the biopsy is estimated to diagnose breast cancer in 4 of these 15 women [3].

MicroRNAs (miRNAs) are approximately 22-nucleotide long RNAs that inhibit gene expression by binding to target messenger RNAs (mRNAs) [4]. Currently, more than 2000 mature human miRNAs have been identified, and these miRNAs may regulate up to 60% of human protein-coding genes [5]. miRNAs are involved in multiple biological processes including cell proliferation, differentiation and apoptosis [6, 7]. Their expression is modified in various cancer subtypes, where these miRNAs act as tumor suppressors or oncogenes and play a key role in tumorigenesis [8].

All cell types release miRNAs in peripheral blood under both normal and pathological conditions. These circulating miRNAs are wrapped in 40-to 100-nm lipoprotein vesicles called exosomes, which are membrane-enclosed cell fragments [9]. These miRNAs appear to be protected from endogenous RNase activity by exosomes and are therefore particularly stable in plasma [10]. Therefore, circulating miRNAs are promising biomarkers for the early and minimally invasive diagnosis of breast cancer [11]. Several studies have already explored miRNAs from that perspective, leading to mixed results in terms of performances [12–29]. Very different diagnostic signatures have been obtained, most likely due to the choice of the sample preparation, the technology used and the study design, such as choice of proper normalization and careful validation.

In the present study, to propose new tools for breast cancer screening, we constructed a diagnostic test based on 8 circulating miRNAs and confirmed its performance in a large cohort of primary breast cancer patients and controls. The diagnostic test was also validated in patients with breast cancer in remission, patients with metastatic breast cancer and patients with gynecologic cancer to test for breast cancer specificity and follow-up. Moreover, particular attention was given to normalization and bioinformatic analysis procedures.

## RESULTS

### Patients and controls

Patients with treatment-naive primary breast cancer (*n* = 149, median age = 55 yr, range = 26–87 yr), breast cancer in remission (*n* = 35, median age = 49 yr, range = 28–79 yr, median time follow-up since remission = 33 months), metastatic breast cancer (*n* = 31, median age = 59 yr, range = 35–79 yr) and gynecologic cancer (*n* = 30, median age = 62 yr, range = 38–83 yr) were recruited prospectively at CHU of Liège and Clinic Saint-Vincent (Liège, Belgium) from 7/2011 to 9/2014. Gynecologic tumors consisted of non-metastatic endometrial (*n* = 16), ovarian (*n* = 10) and cervical (*n* = 4) cancers. Controls were obtained from 133 cancer-free females of similar age (median age = 51 yr, range = 40–74 yr) with normal mammograms (*n* = 72), benign calcifications (*n* = 30) or simple cysts (*n* = 31). Controls had no history of cancer in the last 5 years.

In total, 378 patients were included in this study.

All breast cancer patients and tumor characteristics are summarized in Table 1.

**Table 1:** Clinicopathological data and tumor characteristics

Characteristics	Primary breast cancers – pilot study (*n* = 18)	Primary breast cancers – principal study (*n* = 149)	Metastatic breast cancers (*n* = 31)	Breast cancers in remission (*n* = 35)
**Median age (range) (y)**	58 (29–70)	55 (26–87)	59 (35–79)	49 (28–79)
**Estrogen receptor [*n* (%)]**	12 (67)	117 (79)	28 (90)	22 (63)
**Progesterone receptor [*n* (%)]**	11 (61)	109 (73)	22 (71)	18 (51)
**HER2 [*n* (%)]**	6 (33)	30 (20)	6 (19)	18 (51)
**Ki67 (median ± SD) (%)**	21 ± 20	20 ± 24	27 ± 23	37 ± 23
**Initial T staging [*n* (%)]**				
**NA**	0 (0)	1 (< 1)	2 (6)	0 (0)
**1**	3 (17)	62 (42)	9 (30)	3 (9)
**2**	10 (55)	58 (39)	12 (19)	19 (54)
**3**	2 (11)	15 (10)	6 (19)	5 (14)
**4**	3 (17)	13 (9)	2 (6)	8 (23)
**Lymph node involvement [*n* (%)]**	11 (61)	70 (47)	17 (55)	24 (69)
**Tumor node metastasis (TNM) stage [*n* (%)]**				
**NA**	0 (0)	1 (< 1)	0 (0)	0 (0)
**1**	2 (11)	45 (30)	0 (0)	0 (0)
**2**	9 (50)	73 (49)	0 (0)	20 (57)
**3**	7 (39)	31 (21)	0 (0)	15 (43)
**4**	0 (0)	0 (0)	31 (100)	0 (0)
**Scarff-Bloom-Richardson grade [*n* (%)]**				
**NA**	0 (0)	1 (< 1)	4 (13)	0 (0)
**1**	0 (0)	7 (5)	4 (13)	0 (0)
**2**	7 (39)	84 (57)	12 (39)	15 (43)
**3**	11 (61)	57 (38)	11 (35)	20 (57)
**Histologic subtype [*n* (%)]**				
**NA**	0 (0)	0 (0)	2 (6)	0 (0)
**IDC**	16 (88)	125 (84)	22 (71)	33 (94)
**ILC**	1 (6)	19 (13)	7 (23)	2 (6)
**Others**	1 (6)	5 (3)	0 (0)	0 (0)
**Lymphovascular invasion [*n* (%)]**	6 (33)	27 (21)	12 (39)	9 (26)

NA = not assessed; ER = estrogen receptor; PR = progesterone receptor; HER2 = human epidermal growth factor 2; IDC = invasive ductal carcinoma; ILC = invasive lobular carcinoma.

### Pilot study

A pilot study that consisted of measuring the expression of 742 plasma miRNAs in 18 primary breast cancer patients was first conducted. In total, 188 miRNAs were chosen based on their expression levels (mean quantification cycle (Cq) value < 36) in the pilot experiment. Clinicopathological data for these patients and the list of the 188 selected miRNAs are summarized in Table 1 and Supplementary Table 1, respectively.

### Evaluation of hemolysis

We first evaluated the quality of our sample collection and preparation. Hemolysis leads to the contamination of plasma with RNA from red blood cells. Absorbance at 414 nm (ABS_414_), the maximum absorbance of hemoglobin, correlates with the degree of hemolysis. ABS_414_ was measured for all samples using a NanoDrop. The median ABS_414_ level was 0.19 ± 0.1, with a hemolysis cut-off value fixed at 0.2. Furthermore, the level of a miRNA highly expressed in red blood cells (miR-451) was compared with the level of a miRNA unaffected by hemolysis (miR-23a), with a ΔCq (miR-23a - miR-451) of more than 5 indicating possible erythrocyte miRNA contamination. The median ΔCq (miR-23a - miR-451) was 2.6 ± 1.5 in our cohort (primary breast cancer group = 3 ± 1.5, control group = 2.1 ± 1.2, breast cancer in remission group = 2.5 ± 1.5, metastatic breast cancer group = 2.8 ± 1.2, gynecologic cancer group = 2.3 ± 1.8). Based on these results, no patients were discarded.

### miRNA deregulation is observed in primary as well as metastatic breast cancer patients

When comparing the miRNA profiles of newly diagnosed primary breast cancers to control miRNA profiles, 112 miRNAs were found to be significantly deregulated, with a final set of 107 miRNAs after adjusting the *P*-value for multiple testing. miR-16 and let-7d were the most up- and downregulated miRNAs, respectively. Global upregulation of miRNA expression was observed in primary breast cancer patients compared to controls (1.35-fold change).

In a second analysis, miRNA profiles from the plasma of patients with metastatic breast cancer were compared to those of the controls. Eighty-four miRNAs were found to be significantly deregulated, with a final set of 53 miRNAs after adjusting the *P*-value for multiple testing. The most significantly upregulated miRNA was miR-148a, and the most significantly downregulated miRNA was miR-15b. As observed in primary breast cancer samples, global upregulation of miRNA expression was observed in metastatic breast cancer patients when compared to healthy subjects (1.1-fold change).

Statistical analyses were also performed to compare both primary and metastatic breast cancer patient plasma miRNA profiles to controls using the Kruskal-Wallis test. Fifty-six miRNAs were significantly modified in the same manner among primary and metastatic breast cancer patient profiles. miR-16 and let-7d were the most co-deregulated miRNAs.

The results of the statistical analysis are available in Supplementary Table 1.

### Design and validation of a diagnostic miRNA signature-based model

The analysis and computational methods relied on several steps, which made use of the random forest algorithm. The random forest algorithm is a supervised learning method that operates by building a large ensemble of decision trees, where each tree is trained on a bootstrap sample from the training data by randomizing the features that are selected at each tree node [30].

**Figure 1:**
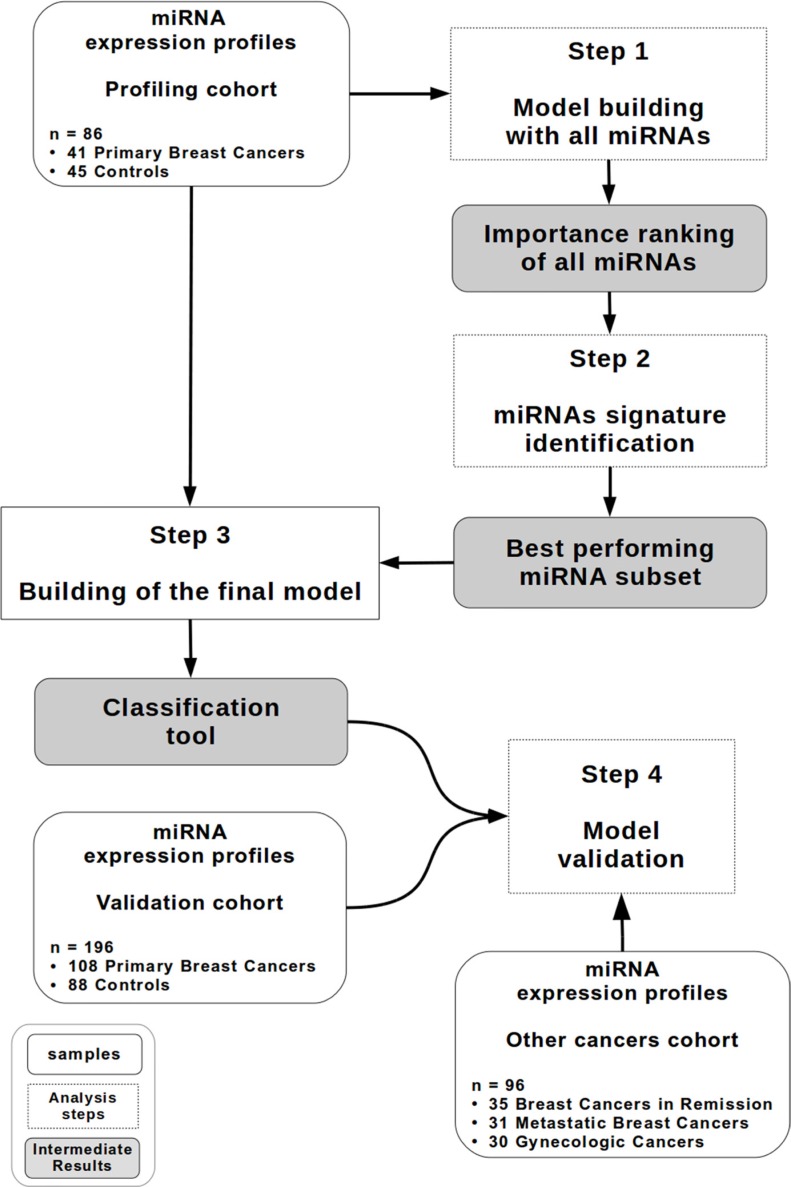
Study design A diagram describing the random forest-based methodology. The profiling cohort (*n* = 86) contains 41 patients with primary breast cancer and 45 controls. The validation cohort (*n* = 196) contains 108 patients with primary breast cancer and 88 controls. The other cancer cohort (*n* = 96) contains 35 patients with breast cancer in remission, 31 patients with metastatic breast cancer and 30 patients with gynecologic cancer.

A methodology somewhat similar to the algorithmic solution proposed by Geurts *et al.* [31] was used as shown in Figure 1.

#### 1 Model construction and miRNA signature identification

An initial random forests model was built on the profiling cohort (86 samples = 30% of the whole cohort: 41 individuals with primary breast cancer and 45 controls) with the normalized expression values of all 188 miRNAs as features to determine the 25 more discriminant miRNAs. To identify the best miRNA signature, all combinations of miRNAs that can be defined from these 25 miRNAs (33554431 in total) were then evaluated using ten-fold cross-validation on the same profiling cohort (see Materials and methods).

**Figure 2:**
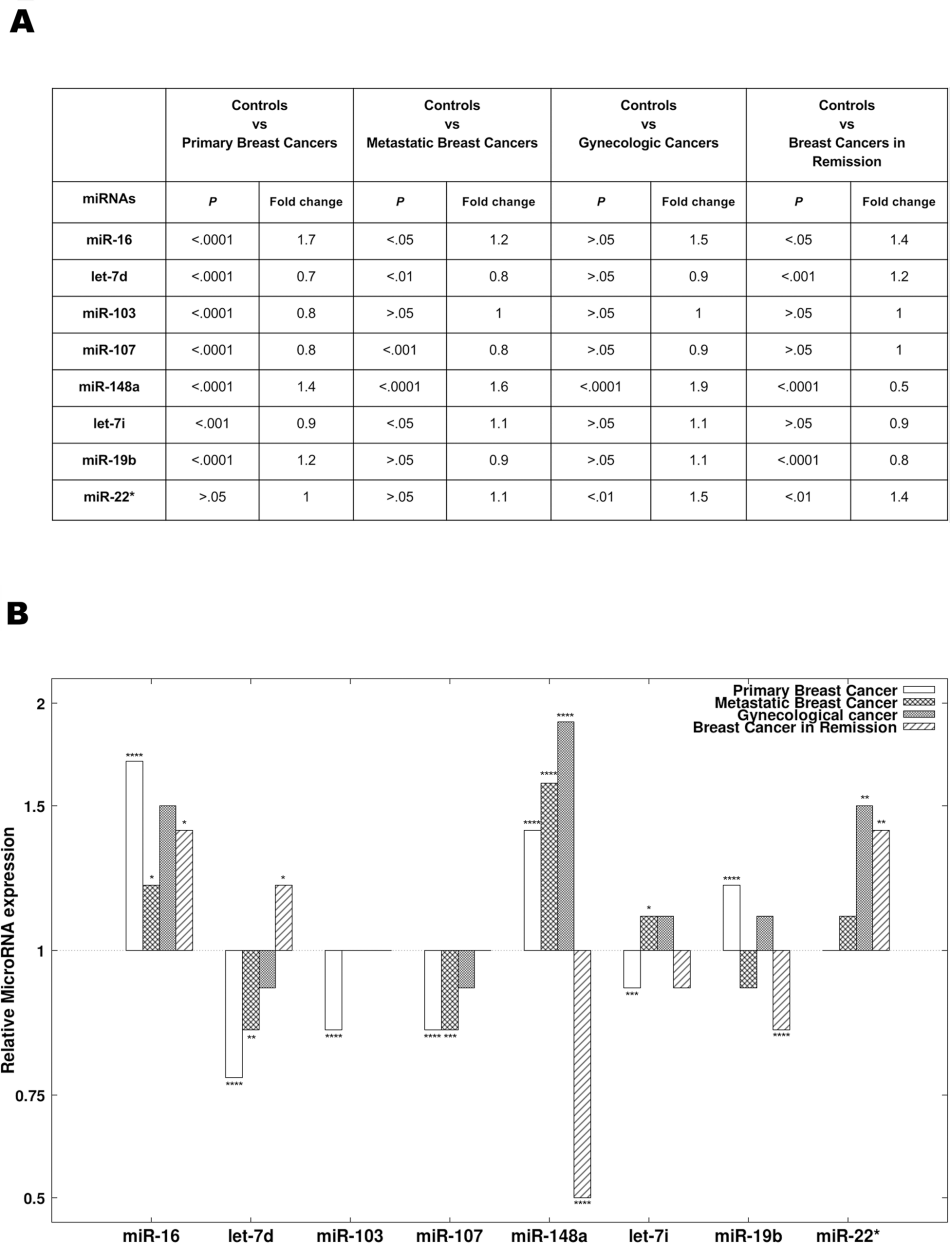
The 8 miRNAs present in the diagnostic signature **(A)** The results of statistical analyses comparing the expression of the 8 miRNAs present in the diagnostic signature between different groups. The 8 diagnostic miRNAs were compared between primary breast cancer patients, breast cancer patients in remission, metastatic breast cancer patients, gynecologic cancer patients and the controls. *P*-values and Benjamini-Hochberg adjusted *P*-values were obtained using the Mann-Whitney *U* test. **(B)** The relative expression (mean fold change) of the 8 diagnostic miRNAs in patients with primary breast cancer, patients with breast cancer in remission, patients with metastatic breast cancer and patients with gynecologic cancer compared to controls.

The best miRNA combination is composed of the following 8 miRNAs: miR-16, let-7d, miR-103, miR-107, miR-148a, let-7i, miR-19b, and miR-22*. Figure 2 summarizes the Mann-Whitney U *P*-values (Figure 2A) and relative expression changes (Figure 2B) for these 8 miRNAs.

An area under the curve (AUC) of 0.85 ± 0.02 was obtained when performing the ten-fold cross-validation in the profiling cohort.

A threshold value of 0.68 was chosen to derive a diagnostic rule from the random forest model. The value of 0.68 corresponded to an acceptable trade-off between high sensitivity (> 0.9) and satisfactory specificity (± 0.5).

#### 2 Model validation

**Figure 3:**
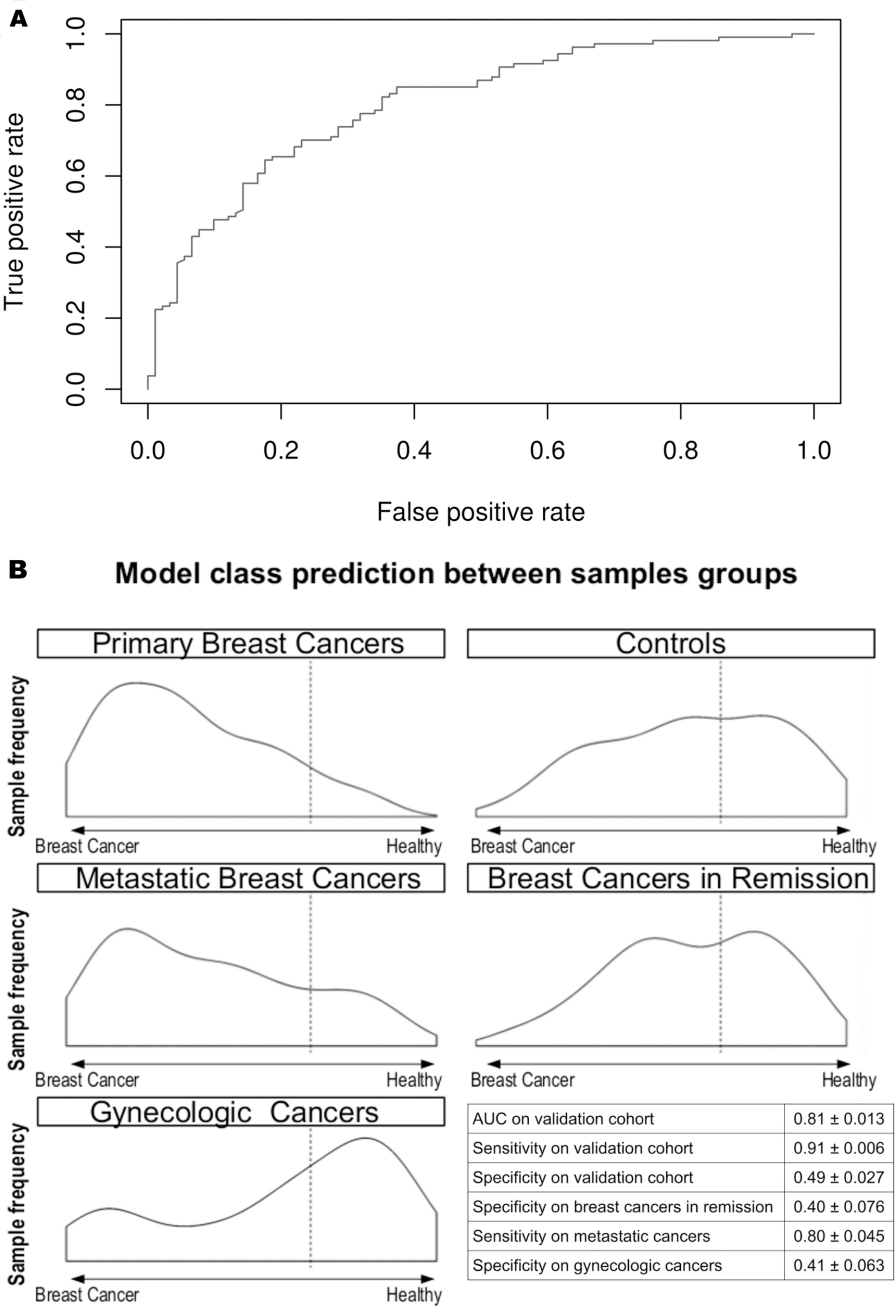
Circulating miRNA-based diagnostic tool performance in the validating cohort **(A)** The ROC curve of the diagnostic miRNA model applied to the validating cohort. The AUC obtained is 0.81. **(B)** Model outcome distributions for the primary breast cancers, controls, metastatic breast cancers, breast cancers in remission, and gynecologic cancers. The x-axis corresponds to the model predictions. The dashed line represents the chosen threshold used to compute the sensitivity and specificity values for each cohort. The table reports the AUC, sensitivity and specificity in the validation cohort and the sensitivity and specificity in the other cancer cohort. The true positive count for the metastatic breast cancers is 25. The true negative count for breast cancers in remission and gynecologic cancers is 14.

The validation of our model in a larger cohort (196 samples = 70% of the whole cohort: 108 individuals with primary breast cancers and 88 controls) yielded an AUC of 0.81 ± 0.01. Figure 3A represents the receiver operating characteristic (ROC) curve obtained by testing the model in the validation cohort.

With a threshold value of 0.68, a sensitivity value of 0.91 ± 0.01 and a specificity value of 0.49 ± 0.03 were obtained.

The validation of the classification model in the other cancer groups yielded slightly lower values for sensitivity (0.80 ± 0.05 for metastatic breast cancer patients) and specificity (0.40 ± 0.08 for breast cancer patients in remission and 0.41 ± 0.06 for gynecologic cancer patients) (Figure 3B). As shown in Figure 3B, the patients with breast cancer in remission and gynecologic cancer were classified as the control group.

### A comparison between the miRNA signature and the established diagnostic methods

Next, we sought to compare the performance of the miRNA signature to mammographic screenings and CA15.3 assays.

**Figure 4:**
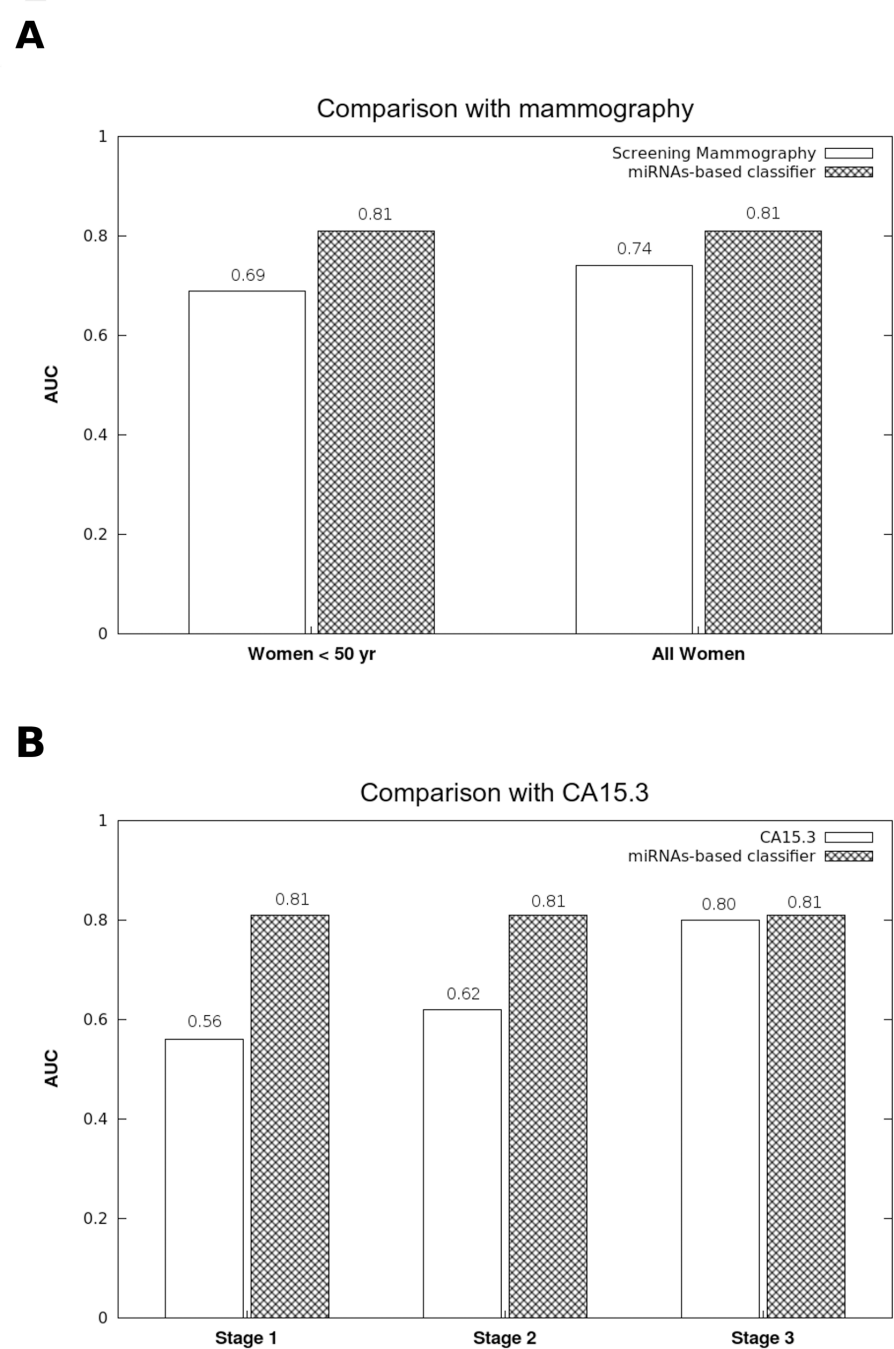
Comparison of the accuracy between the diagnostic miRNA signature, mammographic screenings and CA15.3 assays **(A)** While the diagnostic performance of mammographic screenings is weaker in women under 50 yr (32), the AUC of the 8 miRNA-based diagnostic model was stable for women both under and over 50 yr. **(B)** The CA15.3 assay is not useful for the early diagnosis of breast cancer. While the CA15.3 AUC increases proportionally to the tumor stage (33), our model performance was stable regardless of the tumor stage.

The accuracy of mammographic screening is greatly affected by age. Indeed, young women have dense breasts, making the interpretation of mammography more difficult (AUC = 0.69 ± 0.05 for women under the age of 50 yr) [32]. As shown in Figure 4A, the diagnostic accuracy of the miRNA signature does not appear to be affected by age because the AUC remains stable at 0.81 in patients younger than 50 yr.

CA15.3 is the only biomarker of breast cancer, and its accuracy is directly influenced by tumor stage, with an AUC ranging from 0.56 in stage I to 0.80 in stage III breast cancers [33]. Therefore, CA15.3 is only useful for the diagnosis of late stage and metastatic breast cancers. Interestingly, tumor stage does not seem to affect the signature miRNA performance, remaining stable at 0.81 from stages I to III (Figure 4B).

### miRNA signature does not correlate with breast cancer clinicopathological features

The correlations between the expression of the 8 miRNAs and the following breast cancer clinicopathological markers were computed: estrogen and progesterone receptor expression, HER2 overexpression, tumor size, initial lymph node status, Ki67 index, Scarff-Bloom-Richardson grade and lymphovascular invasion. No significant correlation was obtained using Spearman's test for continuous variables, and no significant difference was found using the Mann-Whitney *U* test for binary variables (Supplementary Table 2).

## DISCUSSION

Early breast cancer diagnosis is currently possible using mammographic screenings. However, mammographic screening has the following weaknesses: (i) the risk of false positives, with an overdiagnosis rate of up to 19%, exposing women to harmful anti-cancer therapies and affecting their quality of life; (ii) the risk of false negatives, with mammograms missing breast cancer in 17% of cases and in more than 30% of cases for women with dense breasts and for women under hormone replacement therapy; (iii) X-ray radiation from mammograms may be one of the factors that can actually trigger breast cancer in high-risk women, e.g., young women carrying a mutation in the BRCA genes, who require early follow up beginning at 30 years, an age where mammography is less effective, and (iv) mammography performance is operator dependent (34–36).

Thus, a diagnostic test using a blood sample could add useful information. CA15.3, which is the only available biomarker for breast cancer, lacks sensitivity in the case of primary breast tumors [33].

Based on 8 circulating miRNAs, we designed a classification model using a decision tree-based ensemble method, which allows primary breast cancers to be screened with greater accuracy than mammography. Consequently, our 8 circulating miRNA signature may be extremely useful to help clinicians to identify patients with a high probability of breast cancer without using invasive procedures.

The 8 miRNA-based diagnostic model shows the following interesting characteristics for clinical application: (i) this diagnostic test is not affected by age and may be useful for monitoring young women at high risk for breast cancer, in which mammography is not only less effective but also harmful because of irradiation; (ii) unlike CA15.3, this diagnostic model is effective regardless of tumor stage, which allows for detection at an early stage; (iii) this model can detect metastatic breast cancers and shows approximately the same class prediction distribution for breast cancers in remission and for controls (see Figure 3), offering a potential utility for monitoring patients; (iv) this study is the first to validate the robustness of such a classifier tool with respect to gynecologic cancers. Plasma from patients suffering of other prevalent cancers in women (cervix, endometrial and ovary cancers) [1] were used to check if the diagnostic tool could avoid the detection of other types of cancers. Indeed, the test specificity on gynecologic cancers is similar to the specificity of the control group (see Figure 3).

These aspects were overlooked in previous studies that have shown the potential of circulating miRNAs as diagnostic tools for breast cancer detection [12–29]. The signatures that these studies have defined differed greatly from one study to another. These discrepancies can be explained by the use of different analysis methods, sample processing and normalization procedures. In the present paper, we show that the appropriate use of a subset of miRNAs combined with a specific normalization method and classification algorithm yields satisfactory results in multiple cohorts. Although decision tree ensemble methods have been proven to be efficient for the classification of biological samples based on various biomarkers [31], to our knowledge, few studies, and never in the field of breast cancer, have used random forest models with miRNA expression values as input features. Two similar studies have nevertheless shown that random forest perform better than other supervised learning methods using miRNA expression values [37, 38].

A second important concern is the normalization choice because the results of the relative quantification obtained by qPCR are entirely dependent on this process. Most of these studies used miR-16 expression alone as a reference gene [20, 25, 28, 29]. However, miR-16, which is predominantly derived from erythrocytes, has been shown to be prone to artificial elevation by hemolysis [18]. The use of blood cell-derived miRNAs as housekeeping RNA for normalization may be more problematic in cases of anemia, a condition often occurring in breast cancer patients. Meanwhile, global normalization methods have been described to best fit with qPCR analysis [39] but to lead to poor performances in discriminating healthy and cancer patients [17]. In this study, we compared different normalization methods, revealing that the mean of the 50 most expressed plasma miRNAs is more stable than many other normalization methods and allows for good discriminating performances. Interestingly, using this method, our analyses revealed that miR-16 and miR-103, which have been used in other studies as endogenous control genes, are differentially expressed in the plasma from healthy samples and cancer patients [12, 21].

Most of the 8 miRNAs that are part of the diagnostic signature are related to well-described cancer deregulation and were demonstrated to be differentially expressed in breast cancer tumoral tissues [40–44]. However, circulating miRNAs rarely show correlated levels with their tumoral expression [26]. In consequence, the miRNA composition of the diagnostic signature does not allow any conclusion on their biological functions.

Aside from the 8 miRNAs selected for our signature, several other combinations, most of which were composed of more than 8 miRNAs, yielded comparable performances. This finding can be explained by the fact that several miRNAs are often deregulated in the same manner under certain conditions, thus allowing one miRNA to be replaced by another miRNA in a specific signature. Regarding independent validation, it can be noted that, among these alternative combinations, one in particular was made of 11 miRNAs, which were measured in the serum of 54 individuals in another independent study [12]. The performance of a diagnostic model built using this alternative combination has been assessed using both our data (plasma) and the dataset GSE42128 from Chan *et al.* (serum), yielding close results (respective AUCs of 0.80 ± 0.02 and 0.77 ± 0.07, see Supplementary Table 3). Unfortunately, one of the miRNAs present in our original signature is absent from the data from Chan *et al.*, preventing us from testing the original signature.

Regarding the potential prognostic value of the 8-miRNA signature, the available follow-up of the cohorts is insufficient to determine whether the expression of the miRNAs can be correlated with progression-free or overall survival. Since there is no correlation between the expression of the 8 diagnostic miRNAs and the currently used clinicopathological factors of breast cancer, the prognostic role of the miRNA signature cannot be established on that base.

In conclusion, we established an accurate miRNA-based model for the non-invasive screening of primary breast cancer. This model also allows the identification of metastatic breast cancer and the classification of breast cancer patients in remission in the healthy group and therefore may be useful for monitoring patients. Moreover, the performance of this test is not affected by the age of the patient or by the tumor stage.

## MATERIALS AND METHODS

### Ethical concerns

Ethic approval was obtained from the Institutional Review Board (Ethical Committee of the Faculty of Medicine of the University of Liège) in compliance with the Declaration of Helsinki. All patients signed a written informed consent form. This work consisted of a prospective study and did not lead to any changes in the treatments of enrolled patients.

### Plasma samples

Blood samples were withdrawn in 9 ml EDTA tubes. Plasma was prepared within 1 h by retaining the supernatant after double centrifugation at 4°C (10 min at 815 × *g* and 10 min at 2500 × *g*) and was stored at −80°C. The absorbance at 414 nm (ABS_414_) was measured for all samples using a NanoDrop to evaluate the degree of hemolysis.

### RNA extraction and miRNA qRT-PCR

The essential MIQE guidelines were followed during specimen preparation [45].

Circulating miRNAs were purified from 100 μl of whole-plasma using a miRNeasy Mini Kit (Qiagen, Germany) according to the manufacturer's instructions. The standard protocol was modified based on Kroh's recommendations [46]. MS2 (Roche, Belgium) was added to the samples as a carrier, and cel-miR-39 and cel-miR-238 were added as spike-ins. RNA was eluted in 50 μl of RNase-free water at the end of the procedure.

Reverse transcription was performed using a miRCURY LNA™ Universal RT microRNA PCR, Polyadenylation and cDNA Synthesis Kit (Exiqon, Denmark). Quantitative PCR was performed according to the manufacturer's instructions on custom panels of 188 selected miRNAs (Pick-&-Mix microRNA PCR Panels, Exiqon). Controls included the reference genes described in the text, inter-plate calibrators in triplicate (Sp3) and negative controls.

All PCR reactions were performed using an Applied Biosystems 7900HT Real-Time PCR System (Applied Biosystems, USA). miRNAs with Cq values < 36 were considered for analysis.

### Data analysis

Analyses were conducted using the 2^−ΔCq^ method (ΔCq = Cq_sample_ – Cq_reference gene_) for each sample to obtain a normalized expression value [47].

The data were normalized using the ΔCq method as recommended by Mestdagh *et al.* [39]. The mean Cq of the 50 miRNAs with the highest mean expression as determined in all the patients from all the cohorts was used for normalization because it was the most stable reference gene according to the GeNorm software. The list of the 50 miRNAs and the results of the GeNorm analysis are available in Supplementary Table 4. The whole processes of miRNA signature identification and decision tree building were also conducted on datasets normalized by 12 alternative methods. The best performances were obtained with the normalization by the mean Cq of the 50 most expressed miRNAs. The alternative normalization were: raw data, mean Cq of the 10, 20, 30 or 40 miRNAs with the highest mean expression, the mean Cq of the 50 miRNAs with the highest mean expression minus the four miRNAs that are present in the signature; the mean Cq of the spike-cel-miR-39 and the U6 small RNA; the mean Cq of miR-15b* and miR-125b (the most stable combination according to NormFinder); the global mean Cq; miR-16; the mean Cq of miR-103 and miR-191; and miR-93.

Furthermore, the delta Cq (miR-23a - miR-451) was determined for each sample to evaluate the risk of hemolysis as recommended by Blondal *et al.* [48].

Finally, data homogeneity was tested to detect outliers. Patients presenting extreme values (mean ± 3 sigma) were discarded. This operation led to the elimination of one patient from the analysis.

Statistical analyses were performed with *R version 3.0.1* (R Core Team (2012). R: A language and environment for statistical computing. R Foundation for Statistical Computing, Vienna, Austria. ISBN 3–900051–07–0, URL: http://www.R-project.org/). To compare miRNA expression levels, two-sided Mann-Whitney *U* tests and Kruskal-Wallis one-way tests were used. To correlate the expression of the 8 diagnostic miRNAs and the clinicopathological markers in primary breast cancer patients, Spearman's tests were used for continuous variables. Statistical significance was established as *P < 0.05 **P < 0.01 ***P < 0.001 or ****P < 0.0001. All represented values were adjusted for multiple testing using the Benjamini-Hochberg procedure [49]. The results of the statistical analyses for selected miRNAs are summarized in Supplementary Tables 1 and 2.

### Study design

For all steps of the method, an *R* implementation of Breiman's original random forest algorithm, which was provided in the *R* package *randomForest*, was used [50]. A methodology somewhat similar to the algorithmic solution proposed by Geurts *et al.* was used [31] as shown in Figure 1. The different steps are described in detail below.

#### 1 Model building with all miRNAs

An initial random forests model was built on the profiling cohort (86 samples: 41 individuals with primary breast cancer and 45 controls) with the normalized expression values of all 188 miRNAs as features. A conservative value of 3000 for *n_tree_* (number of trees in the random forest) was chosen for all steps of the construction of random forest models using our methodology. Because no significant performance change was observed for incremental values of *m_try_* (number of variables randomly sampled as candidates at each split), a default value of $m_{\text{try}}=\sqrt{\text{number of miRNAs}}$ was chosen for all steps of the construction of random forest models using our methodology. A combined ranking for all 188 miRNAs based on the model importance metrics MDA (Mean Decrease in Accuracy) and MDG (Mean Decrease in Gini) was obtained through the construction of this first model.

#### 2 miRNA signature identification

Variable selection in classification or regression methods constitutes a classical problem related to 2 distinct objectives: (i) Finding relevant variables linked to the classifier output, for interpretation purposes (in this case, finding an ensemble of miRNAs related to breast cancer), (ii) Finding a sufficiently small number of variables as to avoid over-fitting, improve model performance, and provide more cost-effective models (both in terms of computation and implementation) [51, 52]. These 2 objectives may often be contradictory, since the first one will be directed to highlighting all important variables, even if these variables are redundant, while the second one aims to limit the number of variables in the final model. We are aiming for the second objective. One variable selection method for random forests, specifically targeting the second objective, is iterative variable elimination [38, 53], where variables with the smallest importance metric are iteratively discarded until reaching a minimum out-of-bag (OOB) error. Based on the definition of MDA provided earlier and the *R* implementation of the random forests algorithm, this feature selection method is roughly equivalent to the iterative elimination of variables with the lowest MDA metric. Another variable selection methodology works the other way round, by iteratively adding variables in candidate models, based on their importance metric, computed on a previous complete model, and stopping the addition of variables when the model accuracy reaches a maximum [31, 54]. Here, we use a more exhaustive wrapper approach, where a large subset of *m* variables is first selected based on the two variable importance metrics (the OOB-related importance metric MDA, but also the Gini coefficient related importance metric MDG) provided by the *R* implementation of the random forests algorithm, and secondly all *c* possible combinations of 1 to *m* variables from this subset are considered as possible features of a potential classifier, where *c* = 2^*m*^ − 1 combinations. This approach thus differs in the fact that it constitutes an exhaustive method, which will test a very large number of combinations. Each of these potential classifiers is cross-validated (with ten folds) to determine the variables combination (also called “signature”) yielding the best performing model (where model performance is measured by the AUC). Since the goal of this study is the design of a usable and affordable diagnostic tool, a limited value of *m = 25* has been chosen (leading to *c* = 33554431). This number corresponds to threshold values of 0.001 and 1 respectively for variable importance metrics MDA and MDG. This limited value of *m = 25* constitutes a trade-off between an exhaustive testing of the solution space and the time and computational limitations related to a diagnostic test.

#### 3 Building the final model

A random forest model was built on the profiling cohort using the best performing miRNA subset. This classification tool constituted the final diagnostic model. The number of trees chosen to build each model was determined as in step 1, and a default value of $m_{\text{try}}=\sqrt{\text{number of miRNAs in the combination}}$ was chosen (i.e. *m_try_* = 3).

The prediction of the random forest algorithm for a sample is a numerical value representing the probability for this sample to be part of a specific class (case or control). To derive a binary diagnostic rule from this numerical score, a specific threshold was picked to separate the 2 classes, and the specificity and sensitivity values of the corresponding rule were computed.

#### 4 Model validation

Then, the classification tool was validated in a larger cohort with similar cases – controls ratio as in the profiling cohort. The total number of samples was 2.3 times greater than profiling cohort (196 samples: 108 individuals with primary breast cancers and 88 controls).

An AUC was obtained through this validation. Sensitivity and specificity values were computed using the threshold defined using the profiling cohort.

The classification tool was also validated in a separate cohort consisting of 35 individuals with breast cancer in remission, 31 patients with metastatic breast cancer and 30 patients with gynecologic cancers.

## SUPPLEMENTARY MATERIALS AND TABLES





## List of abbreviations

3′-UTR: 3′-untranslated region
ABS_414_: absorbance at 414 nm
AUC: area under the curve
Cq: quantification cycle
DNA: deoxyribonucleic acid
gDNA: genomic DNA
HER2: human epidermal growth factor 2
LNA: locked nucleic acid
MDA: mean decrease accuracy
MDG: mean decrease Gini
miRNAs: microRNAs
mRNAs: messenger RNAs
NA: not assessed
Ns: non-significant
OOB: out-of-bag
RNA: ribonucleic acid
ROC: receiver operating characteristic
