# Supplementary material for: Circulating microRNA-based screening tool for breast cancer
Source: Oncotarget. 2015 Dec 29;7(5):5416–28. doi: 10.18632/oncotarget.6786 (PMC4868695; doi:10.18632/oncotarget.6786)
Supplement: Supplementary file 1 [file oncotarget-07-5416-s001.pdf]

Circulating microRNA-based screening tool for breast cancer

Supplementary Materials

Supplementary Table S1: The results of the statistical analyses. To compare miRNA expression levels, two-sided Mann-Whitney *U* tests and Kruskal-Wallis one-way tests were used. All represented values were adjusted for multiple testing using the Benjamini-Hochberg procedure. PBC = primary breast cancer; BCR = breast cancer in remission; MBC = metastatic breast cancer; GC = gynecologic cancer

Supplementary Table S2: The correlations between the expression of the 8 diagnostic miRNAs and the clinicopathological markers in patients with primary breast cancer

	Age	Estrogen receptor expression (positive vs negative)	Progesterone receptor expression (positive vs negative)	HER2 expression (overexpression vs non overexpression)	Size	Initial lymph node status (positive vs negative)	Ki67 index	Scarff Bloom Richardson grade	Lymphovascular invasion (positive vs negative)
***P*-value (Spearman correlation for continuous variables and Mann-Whitney *U* test for binary variables)**									
**miR-16**	> .05	> .05	> .05	> .05	> .05	> .05	> .05	> .05	> .05
**let-7d**	> .05	> .05	> .05	> .05	> .05	> .05	> .05	> .05	> .05
**miR-103**	> .05	> .05	> .05	> .05	> .05	> .05	> .05	> .05	> .05
**miR-107**	> .05	> .05	> .05	> .05	> .05	> .05	> .05	> .05	> .05
**miR-148a**	> .05	> .05	> .05	> .05	> .05	> .05	> .05	> .05	> .05
**let-7i**	> .05	> .05	> .05	> .05	> .05	> .05	> .05	> .05	> .05
**miR-19b**	> .05	> .05	> .05	> .05	> .05	> .05	> .05	> .05	> .05
**miR-22***	> .05	> .05	> .05	> .05	> .05	> .05	> .05	> .05	> .05
**Correlation index (Spearman correlation)**									
**miR-16**	–0.059	NA	NA	NA	–0.15	NA	0.042	0.006	NA
**let-7d**	–0.046	NA	NA	NA	0.046	NA	–0.081	–0.122	NA
**miR-103**	–0.137	NA	NA	NA	0.223	NA	0.002	0.01	NA
**miR-107**	–0.091	NA	NA	NA	0.198	NA	0.08	–0.065	NA
**miR-148a**	0.062	NA	NA	NA	–0.015	NA	–0.055	–0.002	NA
**let-7i**	–0.294	NA	NA	NA	0.125	NA	0.155	0.043	NA
**miR-19b**	0.102	NA	NA	NA	–0.213	NA	0.088	0.063	NA
**miR-22***	0.05	NA	NA	NA	0.017	NA	0.019	0.038	NA

Correlations were calculated using Spearman's test for continuous variables. Comparisons between groups were calculated using the Mann-Whitney *U* test for binary variables.

Supplementary Table S3: Alternative miRNAs signature performances for validation and an independent cohort

Alternative miRNAs combination			
**miR-16 + let-7d + miR-103 + miR-181a + miR-107 + miR-142-3p + miR-148a + let-7f-1 + miR-199a-5p + miR-590-5p + miR-32**			
	AUC	Sensitivity	Specificity
Performances on our dataset (108 primary breast cancers vs. 88 controls)	0.80 ± 0.02	0.9 +–0.04	0.45 +–0.10
Performances with serum on dataset from Chan et al. (GSE42128, 32 primary breast cancers vs. 22 controls)	0.77 ± 0.07	0.9 +–0.10	0.3 +–0.16

Supplementary Table S4: The results of GeNorm analysis

miRNAs	*M* (stability index)
Mean expression of the 50 most expressed miRNAs	0.761
Mean expression of the 120 most expressed miRNAs	0.767
Global mean expression	0.784
hsa-miR-93	0.854
hsa-miR-223	0.860
hsa-miR-425	0.886
hsa-miR-103	0.893
hsa-miR-126	0.899
hsa-let-7g	0.901
hsa-miR-423-3p	0.901
hsa-miR-142-3p	0.922
hsa-let-7d*	0.924
hsa-miR-222	0.924
hsa-miR-484	0.926
hsa-miR-126*	0.926
hsa-miR-26b	0.945
hsa-miR-101	0.951
hsa-miR-15b	0.957
hsa-miR-146a	0.962
hsa-miR-30b	0.963
hsa-miR-191	0.987
hsa-miR-15a	0.990
hsa-miR-652	1.013
hsa-miR-18b	1.017
hsa-miR-21	1.034
hsa-miR-30c	1.035
hsa-miR-148b	1.042

hsa-let-7f	1.046
hsa-miR-125a-5p	1.058
hsa-miR-23b	1.086
hsa-miR-26a	1.091
hsa-miR-145	1.092
hsa-miR-92a	1.140
hsa-miR-221	1.141
hsa-let-7b	1.143
hsa-miR-27a	1.152
hsa-miR-20a	1.156
hsa-miR-486-5p	1.161
hsa-miR-181a	1.168
hsa-miR-19b	1.184
hsa-miR-16	1.191
hsa-miR-23a	1.209
hsa-miR-151-5p	1.221
hsa-miR-320a	1.251
hsa-miR-24	1.291
hsa-miR-199a-5p	1.325
hsa-miR-27b	1.487
hsa-miR-451	1.511
hsa-miR-150	1.637
hsa-miR-199a-3p	1.720
hsa-miR-19a	1.808
hsa-miR-106a	1.844
hsa-miR-107	1.940

GeNorm is an algorithm that determines the most stable reference genes from a set of tested candidate reference genes in a given sample panel. The mean Cq of the 50 most highly expressed miRNAs was used for normalization because it was the most stable reference gene according to the GeNorm software.
